# The Next Generation of Skin Care: Transforming Retinoid Therapeutics

**DOI:** 10.3390/cells14211650

**Published:** 2025-10-22

**Authors:** Julia Weronika Łuczak, Małgorzata Palusińska, Karolina Maślińska-Gromadka, Damian Pietrzak, Tomasz Szopiński, Sławomir Lewicki, Tino Schenk, Łukasz Szymański

**Affiliations:** 1 Department of Molecular Biology, Institute of Genetics and Animal Biotechnology, Polish Academy of Sciences, Postępu 36A, 05-552 Magdalenka, Poland; m.palusinska@igbzpan.pl (M.P.); k.maslinska@igbzpan.pl (K.M.-G.); 2 Department of Nanobiotechnology, Institute of Biology, Warsaw University of Life Sciences, Ciszewskiego 8, Bldg. 23, 02-786 Warsaw, Poland; 3 Chair of Drug and Cosmetics Biotechnology, Faculty of Chemistry, Warsaw University of Technology, Koszykowa 75 Street, 00-662 Warsaw, Poland; damian.pietrzak2.dokt@pw.edu.pl; 4 Institute of Clinical Sciences, Maria Sklodowska-Curie Medical Academy in Warsaw, Pl. Żelaznej Bramy 10, 00-136 Warsaw, Poland; tomasz@urologia.waw.pl; 5 Institute of Outcomes Research, Maria Sklodowska-Curie Medical Academy, Pl. Żelaznej Bramy 10, 00-136 Warsaw, Poland; slawomir.lewicki@uczelniamedyczna.com.pl; 6 Department of Hematology and Medical Oncology, Clinic of Internal Medicine II, Jena University Hospital, 07747 Jena, Germany; tino.schenk@med.uni-jena.de; 7 Institute of Molecular Cell Biology, Center for Molecular Biomedicine Jena (CMB), Jena University Hospital, 07747 Jena, Germany

**Keywords:** retinoids, all-trans-retinoic acid, dermatology, retinoic acid receptors, vitamin A, CYP26 inhibition, skin barrier, synthetic retinoids, microbiome

## Abstract

Retinoids are central regulators of skin biology, influencing keratinocyte proliferation, differentiation, immune modulation, and barrier maintenance. Their therapeutic relevance has long been attributed to retinoic acid receptor (RAR)-mediated transcriptional activity; however, recent studies have revealed additional layers of regulation, including epigenetic modifications, kinase signaling networks, and interactions with the skin microbiome. These mechanisms not only refine our understanding of retinoid function but also inform strategies to overcome therapeutic limitations such as resistance, irritation, and systemic toxicity. Advances in medicinal chemistry have yielded synthetic retinoids with enhanced receptor selectivity, particularly for RAR-γ agonists such as trifarotene, as well as inhibitors of cytochrome P450–mediated retinoic acid metabolism, which sustain endogenous activity and mitigate resistance (DX314 and other RAMBAs). In parallel, the development of nanocarriers, stimuli-responsive gels, and other targeted delivery systems has improved drug stability, bioavailability, and tolerability. Together, these innovations underscore the evolving role of retinoid-based interventions in precision dermatology, providing opportunities to optimize treatment outcomes for acne, psoriasis, photoaging, and other dermatological disorders while addressing the shortcomings of earlier generations.

## 1. Introduction

Retinoids occupy a central role in dermatology due to their ability to modulate essential biological processes, including keratinocyte proliferation and differentiation, immune responses, and extracellular matrix remodeling, critical for maintaining skin homeostasis and barrier integrity. While our previous review comprehensively detailed the foundational understanding of their genomic mechanisms, including retinoic acid receptor (RAR)-mediated transcription and non-genomic pathways, recent research has substantially expanded this view. Beyond canonical RAR/RXR interactions, emerging evidence highlights additional layers of complexity, including epigenetic regulation, kinase signaling crosstalk, and interactions with the skin microbiome, all of which influence retinoid bioavailability and therapeutic efficacy. Endogenous all-trans retinoic acid (ATRA), the biologically active metabolite of vitamin A, is tightly regulated by cytochrome P450 enzymes, particularly CYP26 family members (CYP26A1, CYP26B1, and CYP26C1), which inactivate ATRA to maintain retinoid homeostasis. Dysregulation or overexpression of these enzymes can lead to retinoid resistance, reducing the effectiveness of conventional therapies. Therefore, selective inhibition of CYP26 enzymes has emerged as a promising strategy to elevate endogenous RA levels, enhancing efficacy while minimizing the adverse effects of exogenous retinoid administration. Advances in synthetic retinoids, particularly fourth-generation RAR-γ agonists such as trifarotene, offer improved receptor specificity, efficacy, and tolerability. In parallel, developing advanced delivery systems addresses retinoid instability and systemic toxicity challenges, facilitating targeted delivery and improving patient adherence. Combination therapies integrating retinoids with anti-inflammatory, antimicrobial, or photoprotective agents further enhance therapeutic potential, particularly in resistant or multifactorial skin conditions. Importantly, because retinoid signaling governs fundamental processes of cell differentiation, proliferation, and apoptosis, these mechanisms extend beyond dermatological contexts and are also relevant to oncological research, where retinoids have been explored as modulators of tumor biology and potential therapeutic agents. This review synthesizes these recent developments, emphasizing innovations in molecular targeting, delivery technologies, and translational strategies. Together, these advances provide a framework for next-generation precision therapies that optimize clinical outcomes in acne, psoriasis, photoaging, and other dermatological disorders, while also shedding light on shared molecular pathways that underpin their potential in oncology. The literature discussed in this review was identified through searches in PubMed, Scopus, Google Scholar, and the ClinicalTrials database (NIH, USA), with particular emphasis on publications from 2020 to 2025, providing an updated perspective on topics previously addressed in our earlier work [1].

## 2. Next Generation Synthetic Retinoids

### 2.1. Fourth-Gen RAR-γ Agonists

**Figure 1:**
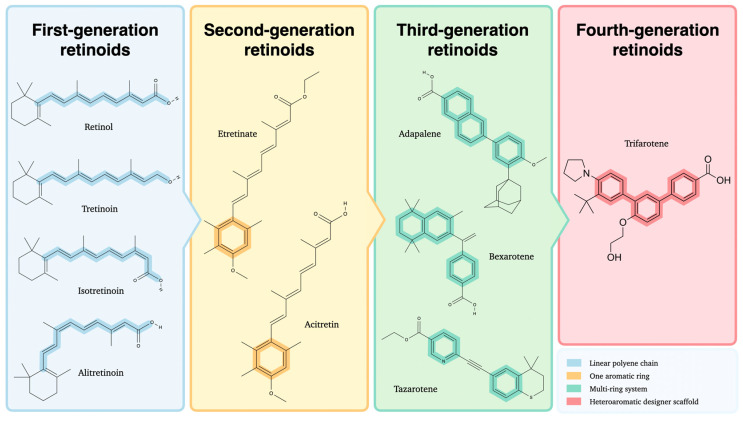
Chemical structures of first-, second-, third-, and fourth-generation retinoids illustrating their structural evolution. Created with GIMP 2.10.

Retinoids are often classified into four generations based on their molecular structure and chemical properties, with representative structural differences depicted in Figure 1 [2].

The first generation consists of naturally occurring retinoids, such as retinol and its metabolites derived from the polyene vitamin A. Numerous studies indicate that these compounds exhibit low specificity in their interactions with retinoid receptors, leading to broad pharmacological effects that may limit their suitability for targeted therapeutic applications [3]. The second generation includes synthetic retinoids, characterized by substituting the cyclohexene ring with a benzene ring or structural alterations mimicking vitamin A [4]. Third-generation retinoids feature a cyclic polyene side chain, which confers selective affinity for retinoic acid receptors β (RAR-β) and γ (RAR-γ), thereby reducing off-target effects compared to earlier retinoids [5]. The most recent developments in the field have focused on synthesizing fourth-generation retinoids, which have been shown to possess an enhanced binding affinity for RAR-γ, particularly within the epidermis [6]. Evidence for epidermal selectivity is strong at the level of receptor binding and distribution, while head to head clinical comparisons with earlier retinoids are not yet available. This characteristic offers a promising prospect for improved therapeutic efficacy and tolerability (Table 1).

The evolution of synthetic retinoid derivatives has been a key focus, aiming to increase receptor selectivity to maximize therapeutic efficacy while minimizing adverse effects. It is evident that traditional retinoids, including tretinoin and isotretinoin, interact with multiple RAR subtypes, which often results in excessive keratinocyte turnover, leading to various side effects such as irritation, peeling, and erythema [13,14]. This lack of receptor specificity has led to selective retinoic acid receptor modulators (RARMs), which are designed to activate RAR-γ, the predominant receptor in epidermal keratinocytes, preferentially. RAR-γ plays a central role in skin homeostasis, regulating keratinocyte differentiation and proliferation while maintaining the structural integrity of the epidermal barrier [15]. By selectively targeting RAR-γ, novel synthetic retinoids enhance therapeutic outcomes in conditions such as acne, psoriasis, and photoaging by significantly reducing irritation and inflammation [16]. Reductions in irritation are biologically plausible, yet they have not been proven against active retinoid comparators in randomized settings. Among these new compounds, trifarotene has emerged as a promising fourth-generation retinoid due to its high specificity for RAR-γ. Clinical studies have demonstrated its effectiveness in reducing acne lesions and improving skin texture with fewer adverse effects than earlier retinoids [15]. The pivotal trials showed strong internal validity but used vehicle controls rather than active retinoids, so relative effectiveness and tolerability versus tretinoin or adapalene remain uncertain [17]. Long term safety data derive from open label cohorts that cannot control for selection and attrition biases [18]. Mechanistic work links retinoid related irritation to TRPV1 activation and to changes in cornified envelope associated proteins, which supports biological plausibility but does not define clinical thresholds for irritation across formulations [14]. Moreover, preclinical research suggests that trifarotene’s selective mechanism minimizes excessive epidermal turnover and inflammation, which are commonly associated with broader retinoid activity [19]. Beyond trifarotene, developing RXR agonists with refined physicochemical properties presents an opportunity to further reduce off-target effects while preserving retinoid therapy’s regenerative and anti-inflammatory benefits [20]. At present, most RXR focused evidence is preclinical or arises from non-dermatology indications, which means dermatology translation will require controlled trials with skin specific endpoints [21,22,23].

The well-established role of RXR agonists in dermatology, as described in our previous work, extends further, with emerging potential applications in immune modulation, oncology, and regenerative medicine [1]. Recent advancements in synthetic RXR ligands have focused on enhancing receptor specificity and improving pharmacokinetics. Notably, bipyridine amide derivatives such as BPA-B9 have shown strong RXRα-binding affinity and selective inhibition of the pRXRα-PLK1 interaction, resulting in potent anticancer activity [24]. Additionally, halogenated retinoid derivatives, including brominated isomers 5a and 5b, have demonstrated selective RARα agonistic activity and RXRα partial agonistic effects, offering therapeutic potential in acute promyelocytic leukemia. These findings indicate mechanistic promise, yet direct dermatology outcomes remain limited, so efficacy in skin disease should be considered preliminary pending clinical testing.

RXR-targeting compounds, particularly RXR agonists, play a crucial role in immune modulation [25]. Combining RXR agonists with immune checkpoint inhibitors has shown potential in enhancing the efficacy of cancer treatments. For example, the RXR agonist LG268 improved the response to anti-PD-L1 therapy in breast cancer models by increasing the infiltration of cytotoxic CD8 T cells [21]. These findings underscore the potential of RXR modulators in combination therapies to improve cancer treatment efficacy while minimizing systemic toxicity. Structural refinement of RXR ligands has led to the development of novel agonists with enhanced selectivity and reduced side effects. Adouvi et al. (2023) demonstrated that combining natural and synthetic ligand features significantly improved RXR agonist potency and stability [22]. Similarly, Lewandowski et al. (2024) leveraged structure-guided drug design to optimize RXR-targeting ligands, resulting in highly potent partial agonists with superior physicochemical properties [23]. These advancements provide a foundation for the next generation of RXR modulators with improved pharmacokinetics and therapeutic potential. However, the RXR literature is dominated by medicinal chemistry and oncology models, so claims of benefit in dermatology should be framed as hypotheses that require confirmation in comparative clinical studies.

Selective RARMs are being explored for their potential applications in regenerative medicine and wound healing. Synthetic RAR ligands have shown promise in enhancing epithelial repair and mitigating oxidative stress, particularly relevant in aging and damaged skin [26]. Additionally, novel RXR agonists have demonstrated the ability to influence immune modulation and epithelial barrier function, offering potential therapeutic avenues for inflammatory skin disorders such as atopic dermatitis and rosacea [27]. However, despite these advancements, challenges remain in optimizing the pharmacokinetic properties of synthetic retinoids to ensure controlled absorption and metabolic stability. It is recommended that future research efforts concentrate on the refinement of ligand structures with a view to enhancing receptor binding affinity whilst concomitantly effecting a reduction in systemic exposure. Such advances have the potential to facilitate the development of next-generation retinoid therapies that exhibit enhanced efficacy, selectivity, and safety. It is imperative to acknowledge that sustained advancement in the domains of ligand engineering and pharmacological evaluation is instrumental in fully actualizing the therapeutic potential of these compounds. In addition, narrative reviews emphasize selectivity advantages but provide limited quantitative synthesis and no head to head comparisons with established retinoids, which lowers certainty in comparative claims [15,19].

Fourth-generation retinoids, particularly selective RAR-γ agonists like trifarotene, offer enhanced receptor specificity, improving therapeutic efficacy in skin disorders while minimizing side effects such as irritation and excessive keratinocyte turnover. Advances in RXR agonists further expand the therapeutic potential of retinoids, including immune modulation and anticancer applications, by enhancing receptor selectivity and pharmacokinetics. Ongoing ligand refinement and pharmacological optimization are crucial for developing next-generation retinoid therapies with superior efficacy, safety, and controlled systemic exposure. Overall, the current evidence supports class effectiveness, while comparative superiority claims for fourth generation agents over established retinoids should be considered provisional until head to head trials are available.

### 2.2. Search Strategies for RXR/RAR Dual Modulators

The pharmacological efficacy and therapeutic selectivity of retinoids are closely determined by their molecular architecture, which governs interactions with nuclear receptors and downstream signaling pathways. Recent advances in computational modeling have substantially improved the elucidation of structure-activity relationships (SARs) in retinoid analogues, offering critical insights into the molecular determinants of their biological activity [28]. By employing in silico techniques such as molecular dynamics simulations, quantum chemical calculations, and molecular docking, researchers have achieved increasingly accurate predictions of ligand–protein interactions [29,30]. These methods accelerate the rational design of synthetic retinoid derivatives and enable preliminary assessments of pharmacodynamic and pharmacokinetic properties before experimental validation. Despite these advances, most medicinal chemistry and in silico reports identify RXR or dual targeting chemotypes without in vivo dermatology endpoints, so efficacy and safety remain hypothetical. A recent, 2025 study exemplified this approach by developing a scalable synthetic platform for 23 novel retinoids and systematically evaluating their receptor interactions. Using the GOLD docking suite with ChemScore, complemented by molecular dynamics simulations, the study demonstrated that subtle modifications of hydrophobic head groups and carboxylate tails markedly influenced binding free energies and isoform selectivity, particularly toward RAR-γ. This underscores the value of SAR-guided in silico strategies in tailoring retinoids with improved specificity while maintaining balanced activity across RAR-α and RAR-β. Alongside these advances in RAR-targeted design, RXR-selective compounds are also emerging. For instance, BPA-B9 has been identified as a potent and selective RXRα antagonist (KD~39 nM), disrupting the RXRα/PLK1 interaction critical for mitotic progression and inducing mitotic arrest in cancer cells. Docking and structural studies confirmed its optimized fit within the RXRα ligand-binding domain, highlighting its therapeutic potential in oncology [31]. Findings on RXR directed agents that modulate antitumor immunity expand mechanistic scope but derive from cancer models, so dermatology applicability requires bridging studies with skin specific outcomes [21]. Concurrent studies concentrated on clinically pertinent compounds, such as trifarotene, utilizing docking protocols that entailed meticulous protein preparation, grid box optimization, and the employment of the Lamarckian Genetic Algorithm in AutoDock (https://autodock.scripps.edu/), predominantly on RAR-α. Although initially directed at this isoform, it is evident that these computational frameworks are readily transferable to RAR-γ. This is due to the fact that, in conjunction with RAR-α, RAR-γ plays a pivotal role in retinoid-mediated transcriptional regulation and is implicated in key processes such as differentiation, immunity, and tumourigenesis. In parallel, the concept of synthetic dual modulators that co-target RAR and RXR subtypes has been proposed as a means to overcome resistance mechanisms such as CRABP-II depletion and CYP26 overexpression in skin diseases, thereby providing more durable therapeutic outcomes [24]. Likewise, selective analogues such as EC23, a photostable benzoic acid derivative, have demonstrated RARβ/γ-specific activity, regulating epidermal proliferation and hair follicle cycling, and showing promise for disorders including alopecia and chronic barrier dysfunction [32]. Expanding such approaches across receptor subtypes, including potential RAR/RXR heterodimer targets, will be essential for advancing SAR-guided design of dual modulators with enhanced therapeutic selectivity. Preclinical strategies that pair RAR engagement with mitigation of retinoic acid catabolism are theoretically attractive, yet superiority over selective RAR-γ agonists remains to be shown in controlled translational studies.

Computational modeling and SAR analyses have advanced the design of retinoid analogues with improved receptor specificity and pharmacological efficacy. In silico strategies, including molecular docking and dynamics simulations, enable rational development of RAR- and RXR-targeted compounds, as well as dual modulators that co-target these receptors to overcome resistance mechanisms. These approaches facilitate the creation of selective retinoids with potential applications in dermatology and oncology, improving therapeutic outcomes while minimizing off-target effects.

### 2.3. Endogenous-RA Boosting via CYP26 Inhibition

ATRA is a pivotal regulator of epidermal differentiation, immune modulation, and barrier integrity. Its homeostasis is tightly controlled by cytochrome P450 enzymes, particularly the CYP26 family (CYP26A1, CYP26B1, and CYP26C1), which metabolize ATRA into hydroxylated inactive forms [33]. Dysregulation or overexpression of CYP26 enzymes has been implicated in retinoid resistance and reduced therapeutic efficacy in various dermatological conditions [34,35,36]. In response to these challenges, a 2020 study introduced DX314, a selective substrate-based inhibitor of CYP26B1 designed to enhance endogenous ATRA levels by preventing its metabolism primarily through this enzyme [16]. Preclinical data demonstrated that DX314 possesses therapeutic potential in keratinization disorders such as congenital ichthyosis and Darier disease, where it was able to preserve epidermal barrier integrity and reproduce the beneficial effects of topical retinoids, while exhibiting minimal off-target activity in both in vitro and in vivo models. Despite compelling pharmacology, randomized human trials that define clinical benefit and dose response for selective CYP26 inhibition in skin are not yet available. The significance of these findings is reinforced by the observation that cytochrome P450 enzymes display tissue-specific expression patterns in the skin. In particular, CYP26A1, CYP26B1, and CYP26C1 contribute critically to the metabolism of endogenous retinoids, thereby ensuring skin homeostasis and proper barrier function. This supports the concept of topical selectivity, although the evidence base remains enzyme level rather than patient level [37]. Understanding these expression dynamics and enzymatic activities is therefore essential for the rational design of topical therapies that modulate retinoid availability and signaling. Further support for this therapeutic approach was provided by Sakamuru et al. (2024), who developed and validated a high-throughput assay to measure CYP26A1 inhibition [38]. By applying this platform, they demonstrated that established retinoic acid metabolism blocking agents (RAMBAs), including liarozole and talarozole, strongly inhibit CYP26A1. Pharmacological blockade of CYP26 increases endogenous ATRA signaling in animal models with tissue specific effects, which complicates dosing strategies and translation to patients [35]. Assay robustness and enzyme mapping are valuable for discovery, yet they provide pharmacologic readouts rather than clinical outcomes. Safety considerations remain salient, since teratology literature indicates that loss of CYP26 activity can increase embryonic retinoid exposure, which underscores the need to minimize systemic absorption and to define risk management frameworks in future studies [34].

CYP26 enzymes tightly regulate ATRA levels by metabolizing it into inactive forms, and their dysregulation can reduce retinoid therapeutic efficacy. Inhibiting CYP26, for example with selective agents like DX314 or RAMBAs, enhances endogenous ATRA concentrations, improving skin barrier function and offering therapeutic potential for keratinization disorders. These findings highlight CYP26 inhibition as a promising strategy for modulating retinoid signaling in dermatological treatments.

## 3. Microbiome Crosstalk

### 3.1. Microbial Retinoid Metabolism

**Figure 2:**
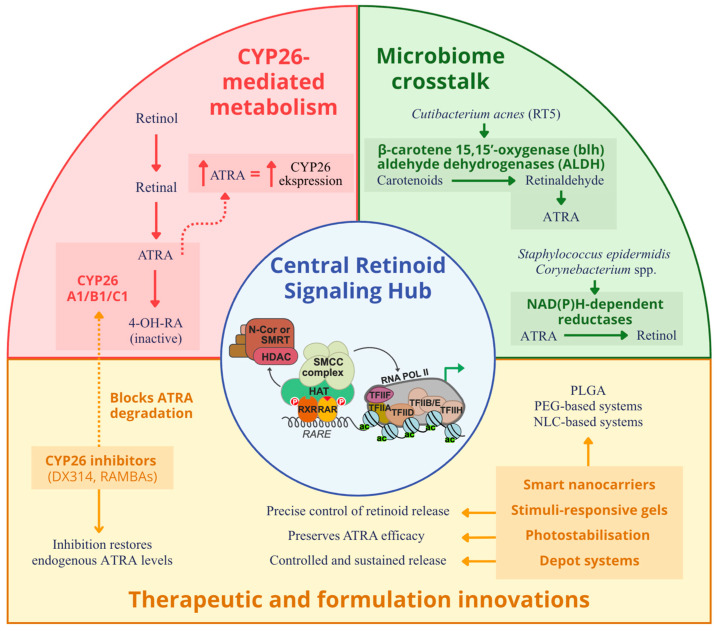
Schematic representation of the interconnected pathways that maintain epidermal ATRA balance. ATRA binds RAR/RXR heterodimers with RAR-γ as the predominant epidermal isoform to regulate genes responsible for differentiation, barrier formation, and immune modulation. ATRA levels are controlled by CYP26A1, CYP26B1, and CYP26C1 enzymes that convert ATRA into inactive metabolites through a negative feedback loop. Inhibitors such as DX314 and RAMBAs block CYP26-mediated degradation and restore endogenous ATRA. Cutaneous microbes modulate retinoid availability: *Cutibacterium acnes* converts carotenoids to ATRA through β-carotene oxygenase (blh) and aldehyde dehydrogenases (ALDH), whereas *Staphylococcus epidermidis* and *Corynebacterium* species reduce ATRA to retinol. Malassezia restricta releases retinyl esters through lipase activity. Smart nanocarriers, hydrogels, and depot formulations enable controlled epidermal delivery and photostabilization, while selective RAR-γ agonists such as trifarotene enhance receptor signaling. Together, host, microbial, and therapeutic factors form a feedback network that sustains retinoid homeostasis in skin. Created with GIMP 2.10.

Cutaneous microorganisms are no longer considered passive bystanders during topical or systemic retinoid therapy. Increasingly, they are recognized as active participants in retinoid bioavailability and signaling modulation. High-resolution metagenomic and culture-based studies have demonstrated that several resident skin taxa encode enzymes capable of interconverting vitamin A derivatives, thus directly shaping local ATRA levels and, by extension, influencing the efficacy and tolerability of retinoid treatments [39]. In particular, whole-genome reconstructions of virulent *Cutibacterium acnes* ribotype five strains have revealed the presence of genes encoding β-carotene 15,15′-oxygenase (blh) and aldehyde dehydrogenases (ALDH), suggesting an intrinsic capacity to convert dietary carotenoids into retinaldehyde and further oxidize retinaldehyde into ATRA. These reactions are predicted, via flux-balance analysis, to be active under lipid-rich, microaerobic conditions characteristic of the pilosebaceous unit, particularly in acne lesions [40]. Complementary in vitro experiments demonstrate that *Staphylococcus epidermidis* and several *Corynebacterium* species possess NAD(P)H-dependent reductases capable of converting ATRA back to retinol. This reductive activity establishes a microbial “sink” that may attenuate local RA signaling and diminish drug efficacy [41]. Recent human studies prove that microbial RA metabolism correlates with therapeutic outcomes. A longitudinal follicular shotgun metagenomic analysis of acne patients undergoing oral isotretinoin therapy showed a marked decline in *C. acnes* RT5 abundance alongside the expansion of RT2 and RT6 strains lacking functional ALDH genes. This strain-level shift correlated with clinical improvement, suggesting that microbial catabolism of RA may serve as a predictive biomarker for treatment response [42]. Beyond *C. acnes*, other skin-associated taxa appear capable of contributing to retinoid interconversion. For instance, *Malassezia restricta* expresses secreted lipases that can release retinyl esters from host-derived sebum triglycerides, supplying substrate for further enzymatic oxidation [43]. These findings are mechanistically plausible, yet much of the evidence is derived from in vitro or in silico systems, so the magnitude of in vivo effects remains uncertain. Moreover, the evaluated study was modest in size and correlative in design, so predictive claims should be considered preliminary until validated in independent populations [42] (Figure 2).

Meanwhile, *Corynebacterium kefirresidentii* has been shown to oxidize retinol to retinaldehyde under aerobic conditions, which may help sustain RA pools in xerotic or aged skin environments [44]. These microbial transformations have notable functional implications. One synthetic retinoid, CD437, has demonstrated dual activity: it exerts bactericidal effects against RA-catabolizing *C. acnes* strains at low micromolar concentrations while acting as a potent RAR-γ agonist in human keratinocytes. This dual mechanism of action underscores the therapeutic potential of designing next-generation retinoids with combined antimicrobial and receptor-selective properties [45]. However, this dual mechanism to clinical benefit in acne has not yet been demonstrated in randomized human studies. Conversely, emerging evidence, largely extrapolated from gastrointestinal and ex vivo skin models, suggests that microbial communities capable of producing ATRA may enhance epithelial immunity. ALDH-positive commensals have been shown to prime retinoid-responsive genes involved in barrier reinforcement and antimicrobial peptide production [46]. These observations support a paradigm in which commensal microbial metabolism of retinoids may either amplify or suppress therapeutic RA signaling depending on the local enzymatic context. Taken together, the microbiome literature supports bidirectional microbe retinoid interactions, while the strength of clinical inference is limited by small sample sizes, heterogeneous methods, and a lack of standardized endpoints [39,40,41,42,43,47].

Skin microbiota actively influence retinoid bioavailability and signaling by converting vitamin A derivatives, thereby modulating the efficacy and tolerability of treatments like ATRA. Specific microbes, such as *C. acnes*, *S. epidermidis*, and *Corynebacterium* species, can both produce and degrade retinoic acid, affecting local RA pools and therapeutic outcomes. These insights highlight the potential for designing next-generation retinoids that combine receptor selectivity with antimicrobial activity, as well as using microbial metabolism as a biomarker for treatment response.

### 3.2. Retinoid–Microbiome Interactions

Host–microbe–retinoid interactions are increasingly recognized as a key determinant of epithelial function, immune regulation, and therapeutic response, as both endogenous and microbial pathways contribute to vitamin A metabolism. Microbial retinoid metabolism relies on conserved enzymatic systems such as alcohol dehydrogenases (ADHs), aldehyde dehydrogenases (ALDHs), retinol dehydrogenases (RDHs), and short-chain dehydrogenases/reductases (SDRs), which convert retinol to retinaldehyde and further to retinoic acid, thereby paralleling host pathways that underpin vision, epithelial homeostasis, and immune balance [48,49,50,51]. Additional enzymes like lecithin-retinol acyltransferase (LRAT) catalyze esterification steps, demonstrating the biochemical overlap between microbial and host retinoid networks [52]. Harnessing these pathways, engineered *Saccharomyces cerevisiae* strains overexpressing transport proteins or human RDH12, as well as *Escherichia coli* strains equipped with β-carotene cleavage enzymes and the mevalonate pathway, have been shown to selectively produce retinol, retinal, or retinoic acid with applications in pharmaceuticals, cosmetics, and nutrition [53,54,55]. These reports focus on yield, stability, and process engineering, with no dermatology therapeutic endpoints, so clinical translation is indirect. Moreover, microbial retinoid production faces challenges including chemical instability, extraction difficulties, and intracellular redox imbalances, which can be mitigated by antioxidants, two-phase fermentation systems, and NADH oxidase overexpression [55,56,57]. Beyond biotechnological use, microbial retinoids play a direct role in host health: deficiencies or dysregulation are implicated in retinal diseases such as age-related macular degeneration, congenital amaurosis, and Stargardt’s disease, as well as in proliferative diseases including cancers and dermatological disorders [58]. It is important to note that, links to cutaneous outcomes are largely inferential and require studies that measure both enzyme activity and clinical endpoints in skin. In dermatology, the host–microbe–retinoid axis is particularly evident in the context of ATRA treatment, where the skin microbiota modulates therapeutic outcomes. ATRA alters the epidermal barrier by regulating tight junction proteins such as Claudin-1 and Claudin-4, while simultaneously shifting microbial diversity—decreasing Staphylococcus while enriching Cutibacterium—changes that correlate with acne improvement but also with barrier disruption [59,60]. In parallel, ATRA reduces Toll-like receptor two expression and modulates cytokine release in keratinocytes, thereby attenuating inflammatory responses to Cutibacterium acnes and enhancing immune tolerance [61]. Moreover, ATRA exhibits direct antibacterial activity against acne-associated microbes, an effect amplified when encapsulated in nanostructured carriers that improve drug stability, reduce side effects such as irritation, and promote barrier repair [62,63]. Clinical studies confirm that such microbiota-mediated and formulation-dependent changes in retinoid dynamics translate into improved outcomes in acne and related skin conditions [47,64]. Taken together, these findings reveal that retinoids act as a central signaling hub at the interface of host and microbes, linking conserved enzymatic pathways to immune and epithelial regulation, while also offering translational opportunities in metabolic engineering and microbiome-informed therapies [54,65].

Interactions between retinoids, the host, and the microbiome play a crucial role in regulating epithelial integrity, immunity, and therapeutic outcomes. Both host and microbial enzymatic pathways contribute to retinoid metabolism, influencing skin health and responses to treatments like ATRA. Understanding these interconnected processes enables the development of microbiome informed and formulation dependent strategies that optimize efficacy and tolerability, while prospective controlled studies are needed to confirm clinical benefit.

## 4. Next Generation Delivery Systems

### 4.1. Smart Nanocarriers and Stimuli-Responsive Gels

The field of drug delivery has undergone a substantial transformation with the emergence of advanced nanoscale delivery systems designed to enhance the specificity, efficacy, and safety of therapeutic interventions. Among the most promising innovations are smart nanocarriers and stimuli-responsive platforms. These have demonstrated significant potential in addressing longstanding challenges associated with conventional drug administration, including poor bioavailability, off-target effects, and systemic toxicity. These next-generation systems are engineered to respond to specific internal or external stimuli, such as variations in pH, redox gradients, enzymatic activity, temperature, magnetic fields, ultrasound, or light, allowing for spatiotemporally controlled release of therapeutic agents at the intended site of action [66]. This level of precision is particularly valuable in the context of treatments requiring tightly regulated pharmacokinetics and targeted biodistribution, such as those involving ATRA. While it can potentially be used in various clinical settings, including oncology and regenerative medicine, it is often limited by rapid metabolism, poor aqueous solubility, and adverse systemic effects. Li et al. (2017) comprehensively discuss the application of polymeric nanoparticles, such as PLGA and PEG-based systems, for improved cancer therapy [67]. These nanocarriers demonstrate enhanced drug solubility, stability, and circulation half-life, as well as targeted accumulation at tumor sites through the enhanced permeation and retention effect. Moreover, PEGylation strategies were shown to reduce hepatic accumulation and prolong systemic circulation, thereby increasing the precision of cancer stem cell targeting. In a recent study, researchers developed a tretinoin-loaded NLC formulation (NLC-TRE) to enhance the safety profile and skin-targeting efficiency of tretinoin (TRE). Utilizing a 2^3^ factorial design, the NLC-TRE was thoroughly characterized (including HPLC, dynamic light scattering, differential scanning calorimetry, X-ray diffraction, and electron microscopy). Compared to a marketed 0.05% TRE cream, the NLC-TRE demonstrated superior oxidative stability and significantly enhanced epidermal targeting in vitro and in vivo. Moreover, safety assessments using reconstructed human epidermis and transepidermal water loss measurements in healthy volunteers confirmed a markedly improved tolerability profile (*p* < 0.0002). These findings indicate that NLC-based systems may provide a safer and more effective alternative for the topical administration of TRE in treating various skin disorders [68]. The human component was short term and focused on tolerability in healthy volunteers, and randomized trials against active comparators are not yet available, so clinical advantage remains provisional. Stimuli-responsive hydrogels represent another innovative approach in retinoid delivery. New formulations that respond to skin pH or temperature changes allow for more precise modulation of retinoid release. Early clinical evaluations suggest these systems reduce irritation while treating photoaging and acne effectively. For instance, chitosan-based injectable hydrogels have been developed that undergo sol–gel transition in response to external stimuli, showing significant advantages in the on-demand release of drug molecules. These hydrogels exhibit excellent stimuli-responsive properties and thixotropic features, including pH-responsive, self-healing, and injectable properties. Additionally, advancements in hydrogel-based drug delivery systems have focused on developing systems more responsive toward changes in external stimuli like pH, temperature, or light for targeted and on-demand drug release [69,70]. Recent advances in polymer chemistry have fabricated hydrogels with improved biocompatibility, mechanical strength, and degradation profiles, yielding various biomedical applications [71]. Moreover, the combination of nanotechnology with hydrogels has rendered new opportunities for drugs and the delivery of complex drugs such as proteins, peptides, and nucleic acids, which are difficult to administer by traditional drug delivery methods. These novel systems are also being explored for localized and sustained drug delivery, especially in cancer therapy. Notably, recent studies have introduced 3D bioprinted hydrogel meshes incorporating ATRA-loaded polymeric particles, which not only enabled a finely tunable and sustained release of the drug but also elicited significant apoptotic responses in U-87 MG glioblastoma cells [72]. Disease biology and dosing paradigms in oncology differ from cutaneous indications, so these results should be viewed as proof of concept rather than directly generalizable. Integrating smart nanocarriers and stimuli-responsive hydrogels offers a multifaceted approach to overcoming the limitations of traditional retinoid therapies. By enabling controlled, targeted, and sustained release of retinoids, these advanced delivery systems hold the potential to enhance therapeutic outcomes while minimizing adverse effects. Continued research and development are essential for translating these promising technologies into clinical practice.

Advances in smart nanocarriers and stimuli-responsive hydrogels have revolutionized drug delivery by enabling precise, controlled, and targeted release of therapeutic agents. These systems enhance the stability, bioavailability, and safety of compounds such as ATRA and tretinoin, addressing challenges like poor solubility and systemic toxicity. Their integration offers a promising strategy for improving treatment efficacy and tolerability in dermatological and oncological applications. However. at present, most dermatology evidence is preclinical or limited to short human tolerability studies, so the strength of inference about superiority over approved formulations is low

### 4.2. Photostabilisation and Depot Systems

ATRA is a pivotal therapeutic agent in dermatology and oncology owing to its pleiotropic biological effects, ranging from regulation of cell differentiation to modulation of immune responses. Nevertheless, its clinical utility is markedly constrained by inherent physicochemical limitations, including rapid photodegradation, poor aqueous solubility, and a short systemic half-life [73]. Consequently, considerable research has been directed toward designing photostabilized formulations and depot delivery systems that can prolong ATRA’s bioactivity, mitigate side effects, and improve patient adherence. In this regard, photostabilization plays a decisive role by protecting ATRA from light-induced breakdown, thereby preserving its therapeutic potency in applications such as photodynamic therapy and dermatological interventions, while simultaneously reducing the risk of treatment failure and recurrence. Complementarily, depot systems provide controlled and sustained drug release, ensuring consistent therapeutic concentrations over extended periods and reducing the frequency of administration, which is particularly advantageous for chronic conditions. Building upon these principles, recent innovations have introduced phospholipid/zein hybrid nanoparticles as biodegradable carriers that substantially enhance ATRA’s photostability. These nanocarriers exploit the amphiphilic characteristics of phospholipids and the structural resilience of zein protein to create a protective milieu, shielding ATRA from photo-induced degradation while enabling controlled drug release. Such systems have achieved average particle sizes below 200 nm and demonstrated significant preservation of ATRA’s bioactivity, with concomitant reductions in cytotoxicity in human cell lines [74]. Parallel advances in depot technologies include azide–alkyne cross-linked hydrogels based on poly(caprolactone-co-lactide)–poly(ethylene glycol)–poly(caprolactone-co-lactide) triblock copolymers, which exhibit thermo-responsive gelation at physiological temperature and allow sustained release of ATRA over approximately two weeks. Notably, these hydrogels display self-healing capabilities, restoring their structural integrity after physical disruption, thereby ensuring a stable and reliable delivery profile [75]. In addition, nanostructured lipid carrier (NLC) composite gels have emerged as potent topical platforms that improve ATRA’s stability and transdermal penetration and mitigate skin irritation through controlled slow release. Preclinical studies in alopecia areata models revealed that ATRA-loaded NLC gels facilitated follicular targeting and stimulated robust hair regrowth by modulating molecular pathways central to hair regeneration [76]. These advances in photostabilized nanocarriers and depot-based delivery modalities effectively circumvent the intrinsic shortcomings of free ATRA, namely instability, rapid clearance, and dose-limiting toxicity. As a result, they substantially enhance its therapeutic efficacy and broaden its translational potential across diverse clinical contexts, ranging from dermatological disorders to highly refractory malignancies.

ATRA, despite its potent therapeutic effects in dermatology and oncology, faces major limitations due to poor stability, solubility, and rapid degradation. Recent advances in photostabilized nanocarriers and depot delivery systems, such as phospholipid/zein nanoparticles, thermo-responsive hydrogels, and nanostructured lipid carriers, have significantly improved ATRA’s stability, controlled release, and bioactivity. These innovations enhance therapeutic efficacy, reduce toxicity, and expand ATRA’s applicability in both dermatological and oncological treatments. However, without randomized patient trials that compare these platforms with approved topical formulations and incorporate rigorous safety monitoring, the strength of inference about clinical superiority remains low

## 5. Clinical Translation—Acne and Truncal Acne

Clinical Translation of Acne and Truncal Acne is a critical area of dermatological research that bridges the gap between experimental laboratory findings and practical therapeutic applications. This translational process focuses on rigorously tested interventions that aim to improve clinical outcomes for patients suffering from facial and truncal acne, conditions marked by their complex pathophysiology encompassing inflammation, follicular hyperkeratinization, sebum production, and microbial colonization. Mechanistic principles discussed above support specific, testable predictions for clinical outcomes. RAR-γ selectivity should concentrate activity in the epidermis, which can reduce off target RAR-α and RAR-β signaling and may limit irritation that is linked to TRPV1 activation and cornified envelope remodeling [13]. Formulation engineering such as nanostructured lipid carriers can improve stability and epidermal targeting, which can influence adherence and tolerability [68,77,78,79]. Microbiome interactions can shift strain composition and innate immune tone, which can modulate response to therapy [39,42,47].

Recent clinical trials registered on ClinicalTrials.gov illustrate the forefront of this endeavor by assessing the safety, efficacy, and long-term sustainability of novel and established treatments. For instance, the pivotal Phase IIb/III study of B244, a topical agent designed to reduce both inflammatory and non-inflammatory acne lesions, demonstrated promising results in subjects with mild to moderate acne vulgaris, highlighting the clinical potential of this novel intervention in achieving significant lesion reduction over a 12-week period (NCT02832063) [80]. This aligns with the concept that modulation of the follicular microenvironment and innate pathways can complement RAR targeted approaches, since isotretinoin response tracks with shifts in *Cutibacterium acnes* strain composition [42]. Complementing this, an open-label, long-term extension study evaluating CB-03-01 cream over 12 months affirms the importance of sustained treatment strategies, as it documented the compound’s tolerability and sustained anti-acne effects on facial and truncal regions (NCT02682264) [81]. Although CB-03-01 is not a retinoid, its use in truncal disease supports a layered strategy in which receptor selective retinoids deliver comedolysis and normalization of keratinization while agents that target sebum and inflammation address complementary axes of pathophysiology.

Specifically addressing truncal acne, the Sarecycline Truncal Acne Safety and Efficacy Response (TASER) clinical trial underscores the evolving pharmacological approach to truncal acne, with Sarecycline, a tetracycline-class antibiotic with anti-inflammatory properties, being evaluated for its targeted efficacy and safety profile in moderate-to-severe cases (NCT05010538) [82]. Antibiotic effects on the skin microbiome vary across studies and can interact with retinoid driven changes in barrier and innate signaling, which reinforces the need to measure strain level shifts alongside clinical endpoints in truncal cohorts. In parallel, RAR-γ selective trifarotene showed efficacy on facial and truncal lesions with strong internal validity, which is consistent with epidermal targeting described earlier, although the use of vehicle control rather than active retinoids leaves relative benefit and tolerability unresolved [15,17,18]. Formulation advances such as tretinoin NLCs demonstrate improved stability and short term tolerability in humans, so future truncal trials should test whether these delivery gains translate into higher adherence and fewer discontinuations compared with approved creams [78,79].

Such focused clinical research reflects a nuanced understanding of anatomical and pathological variances within acne manifestations, emphasizing the need for tailored therapeutic regimens. The academic rigor inherent in these clinical trials extends to methodological considerations, including robust study designs, adherence to ethical standards such as the Declaration of Helsinki, and validated outcome measures to assess both lesion count reductions and quality-of-life improvements. To strengthen the link from mechanism to clinic, upcoming studies should incorporate mechanistic biomarkers that map to the earlier sections, for example TEWL and stinging scores as readouts of TRPV1 linked irritation for RAR-γ agents, corneocyte maturity and differentiation markers for CYP26 modulation, and strain resolved metagenomics for treatments expected to alter *Cutibacterium* ecology. Moreover, the cross-disciplinary collaboration among dermatologists, pharmacologists, and clinical researchers ensures that therapeutic innovations translate effectively from bench to bedside.

In summary, clinical translation in acne and truncal acne constitutes a dynamic interplay of novel drug development, rigorous clinical evaluation, and patient-centered care principles. The comprehensive body of ongoing clinical research, as exemplified by trials like NCT02832063, NCT02682264, and NCT05010538, provides robust evidence to refine and optimize acne management strategies, ultimately advancing both scientific understanding and clinical practice in this prevalent dermatological disorder. At the same time, the quality of evidence remains uneven. Several datasets are open label or single center, and pivotal trifarotene trials used vehicle control rather than active retinoid comparators, which limits inferences about relative benefit and tolerability. Peer reviewed efficacy reporting for B244 is limited, the CB-03-01 extension emphasizes tolerability without a comparator, and the sarecycline truncal study is descriptive, so certainty for comparative conclusions is low [80,81,82]. Standardized truncal outcome measures and longer follow up are needed, together with patient reported outcomes and safety monitoring that enable synthesis across studies. Future trials should prioritize randomized head to head designs against established retinoids and antibiotics and should incorporate stratification by anatomical site and severity, so that clinical guidance can move from class level to regimen specific recommendations.

## 6. Safety, Regulatory, and Translational Considerations

The development of next-generation retinoids has increasingly emphasized safety and translational applicability. By refining receptor selectivity and delivery mechanisms, these agents aim to preserve therapeutic efficacy while mitigating irritation, teratogenicity, and systemic toxicity that have limited earlier compounds. Trifarotene, a fourth-generation and highly RAR-γ–selective agonist, exemplifies these advances. Clinical trials have demonstrated favorable tolerability, with local reactions such as erythema, dryness, or desquamation generally mild and transient [17,18,19]. A 52-week open-label study confirmed sustained tolerability, with adverse events mostly limited to mild application-site reactions [83]. Nevertheless, comparative head-to-head studies with first- or third-generation retinoids remain limited. Teratogenicity continues to represent a major safety concern across all retinoid classes. Although trifarotene exhibits minimal systemic absorption following topical use, animal studies have shown placental transfer and embryotoxicity [84]. Accordingly, it remains contraindicated during pregnancy and lactation, and in women planning conception. Regulatory labeling is consistent with other topical retinoids, reflecting the persistent uncertainty in epidemiological data, which suggest low but unquantified risk. Market authorization for next-generation retinoids requires rigorous demonstration of safety and efficacy, particularly in pediatric and geriatric populations. Trifarotene received FDA approval in 2019 for acne vulgaris in patients aged ≥9 years based on phase III trials (PERFECT 1 and 2) [17], but its long-term safety in younger children and elderly individuals remains unestablished. The absence of direct comparative trials with established agents such as tretinoin or adapalene complicates regulatory positioning and pharmacovigilance assessments. From a translational perspective, novel formulations must not only improve pharmacokinetics but also meet Good Manufacturing Practice (GMP) and reproducibility requirements. Lipid-based nanocarriers and polymeric nanoparticles have shown preclinical promise in stabilizing retinoids, enhancing tissue specificity, and reducing hepatic metabolism [68,77,78]. However, translation remains limited by formulation complexity and evolving regulatory frameworks for nanomedicines [85]. Endogenous analogues such as ATRA are constrained by low aqueous solubility and photolability. Modern nanocarrier and depot systems, solid lipid nanoparticles, nanostructured lipid carriers, and polymeric micelles, address these limitations by providing controlled and sustained release [68]. Such systems have achieved superior epidermal targeting and reduced irritation compared with conventional creams [62]. Nevertheless, only a few retinoid nanoformulations have reached clinical evaluation, reflecting persistent challenges in large-scale manufacturing and long-term safety assessment [79]. Ultimately, successful clinical translation of next-generation retinoids will depend on bridging preclinical innovation with robust clinical validation. Future research should prioritize scalable manufacturing, standardized safety evaluation, and biomarker-driven clinical trials to establish therapeutic superiority and ensure regulatory compliance.

## 7. Conclusions

Recent advances in retinoid research have transformed the therapeutic landscape by integrating developments in receptor biology, enzymatic regulation, microbiome interactions and drug delivery technology. Fourth-generation RAR-γ-selective agonists, CYP26 inhibitors and RXR modulators designed using a rational approach exemplify the shift towards precision-targeted, well-tolerated therapies. Concurrent developments in nanocarriers, depot systems and stimuli-responsive gels have overcome the long-standing issues of instability, irritation and poor bioavailability, while enabling controlled, sustained release. Emerging insights into microbial retinoid metabolism highlight the significance of the skin microbiome in regulating ATRA availability and as a potential biomarker of treatment response. This multidimensional understanding of retinoid action, from receptor signaling to host–microbe crosstalk, provides a framework for developing next-generation interventions that combine pharmacological selectivity with biological adaptability. However, translating these innovations into clinical practice will require rigorous comparative trials, standardized outcome measures and long-term safety data. Ultimately, bridging mechanistic discoveries with regulatory and clinical validation will define the therapeutic potential of next-generation retinoids in dermatology and beyond.

## Figures and Tables

**Table 1:** Generational Classification of Retinoids: Compounds, Mechanisms, Therapeutic Applications, and Limitations.

Generation	Compounds	Strengths	Weaknesses	Therapeutic Uses	References
First Generation Naturally occurring retinoids derived from vitamin A.	Retinol Retinaldehyde Tretinoin Isotretinoin Alitretinoin	- Broad biological activity. - Effective for acne and photoaging - Strong clinical evidence for tretinoin in anti-aging treatments	- Non-selective receptor binding - High irritation potential - Teratogenicity risks	Acne, photoaging, psoriasis, ichthyosis	[3,7]
Second Generation Synthetic analogs with structural modifications	Etretinate Acitretin	- Effective for severe skin conditions like psoriasis. - Longer-lasting effects due to slower metabolism	- High systemic toxicity - Restricted to oral use - Teratogenicity risks	Severe psoriasis, keratinization disorders	[8]
Third Generation Retinoidal benzoic acid derivatives with receptor selectivity	Adapalene Tazarotene Bexarotene	- Improved receptor selectivity (RAR targeting). - Reduced irritation compared to first-generation compounds. - Effective in localized treatments.	- Can still cause irritation in sensitive skin. - Limited systemic applications	Acne (adapalene), photoaging (tazarotene), cutaneous T-cell lymphoma (bexarotene)	[9,10]
Fourth Generation Highly selective retinoids targeting specific receptors (e.g., RAR-γ).	Trifarotene	- High receptor specificity minimizes side effects. - Effective for localized skin conditions	- Limited clinical data due to recent development - Narrow therapeutic applications so far	Acne (trifarotene), emerging uses under investigation	[8,11,12]

## Data Availability

No new data were created or analyzed in this study. Data sharing is not applicable to this article.

## Abbreviations

The following abbreviations are used in this manuscript:

ATRA	All-trans retinoic acid
RAR	Retinoic acid receptor
RXR	Retinoid X receptor
CYP26	Cytochrome P450 family 26 enzymes
RAMBAs	Retinoic acid metabolism blocking agents
RARMs	Retinoic acid receptor modulators
SAR	Structure-activity relationship
ADH	Alcohol dehydrogenase
ALDH	Aldehyde dehydrogenase
RDH	Retinol dehydrogenase
SDR	Short-chain dehydrogenase/reductase
LRAT	Lecithin-retinol acyltransferase
NLC	Nanostructured lipid carrier
PLGA	Poly(lactic-co-glycolic acid)
PEG	Polyethylene glycol
DSP	Digital signal processing
CRABP-II	Cellular retinoic acid-binding protein II
blh	β-carotene 15,15’-oxygenase
